# Smart Scheduling (SMASCH): multi-appointment scheduling system for longitudinal clinical research studies

**DOI:** 10.1093/jamiaopen/ooac038

**Published:** 2022-05-25

**Authors:** Carlos Vega, Piotr Gawron, Jacek Lebioda, Valentin Grouès, Piotr Matyjaszczyk, Claire Pauly, Ewa Smula, Rejko Krüger, Reinhard Schneider, Venkata Satagopam

**Affiliations:** 1 Luxembourg Centre for Systems Biomedicine, Université du Luxembourg, Esch-sur-Alzette, Luxembourg; 2 Institute of Computing Science, Poznan University of Technology, Poznan, Poland; 3 Parkinson Research Clinic, Centre Hospitalier de Luxembourg, Luxembourg, Luxembourg; 4 Transversal Translational Medicine, Luxembourg Institute of Health, Strassen, Luxembourg

**Keywords:** data integration, multi-appointment scheduling problems, web services

## Abstract

**Objective:**

Facilitate the multi-appointment scheduling problems (MASPs) characteristic of longitudinal clinical research studies. Additional goals include: reducing management time, optimizing clinical resources, and securing personally identifiable information.

**Materials and methods:**

Following a model view controller architecture, we developed a web-based tool written in Python 3.

**Results:**

Smart Scheduling (SMASCH) system facilitates clinical research and integrated care programs in Luxembourg, providing features to better manage MASPs and speed up management tasks. It is available both as a Linux package and Docker image (https://smasch.pages.uni.lu).

**Discussion:**

The long-term requirements of longitudinal clinical research studies justify the employment of flexible and well-maintained frameworks and libraries through an iterative software life-cycle suited to respond to rapidly changing scenarios.

**Conclusions:**

SMASCH is a free and open-source scheduling system for clinical studies able to satisfy recent data regulations providing features for better data accountability. Better scheduling systems can help optimize several metrics that ultimately affect the success of clinical studies.

## Lay Summary

Longitudinal clinical research studies require conducting various assessments over long periods of time. Such assessments comprise numerous stages, requiring different resources defined by multidisciplinary research staff and aligned with available infrastructure and equipment, altogether constrained by time. While it is possible to manage the allocation of resources manually, it is complex and error-prone. Efficient multi-appointment scheduling is essential to assist clinical teams, ensuring high participant retention and producing successful clinical studies, directly impacting patient throughput and satisfaction. This application note presents Smart Scheduling (SMASCH) system, a web application for multi-appointment scheduling management aiming to reduce times, optimize resources and secure personal identifiable information. SMASCH is present in multiple clinical research and integrated care programs in Luxembourg since 2017, including Dementia Prevention Program, the study for Mild Cognitive Impairment and gut microbiome, and the National Centre of Excellence in Research on Parkinson’s disease which encompasses the study for REM sleep behavior disorder and the Luxembourg Parkinson’s Study.

## OBJECTIVE

We introduce Smart Scheduling (SMASCH) system, a web-based tool designed for longitudinal clinical studies requiring recurrent follow-up visits of the participants. Longitudinal clinical research studies are often ran on one-stop shop setups in which participants only need to visit the clinical research facility few times to undertake all required assessments with different specialists in the same place. Participants of such studies usually undergo a series of follow-up appointments with a reduced clinical visit frequency (eg, yearly). SMASCH assists clinical teams on the management of sensitive information, required items for conducting assessments, and scheduling participant appointments. Although it does not provide full automatic scheduling of individual appointments, it automatically creates follow-up visits for the participants once the previous visit is finished. Generally, such visits must be consistently spaced in time to prevent misalignment or time lag. In this context, we refer to visit as the date interval in which all required assessments need to be conducted to be considered an individual time-point for the clinical studies.

### Background and significance

Marynissen and Demeulemeester^1^ reviewed multi-appointment scheduling problems in hospitals (MASPHs) and the different methodologies available in the literature to find the optimal assessment paths for patients. Their work shows different MASPH scenarios such as general hospitals or facilities for diagnostic tests.

In this sense, SMASCH is present in multiple clinical research and integrated care programs in Luxembourg since 2017, including Dementia Prevention Program (PDP, http://demence.lu); the study for Mild Cognitive Impairment and gut microbiome (MCI-BIOME); and the National Centre of Excellence in Research on Parkinson’s disease (NCER-PD, https://parkinson.lu),^2^ which encompasses the study for REM sleep behavior disorder (RBD, https://rbd.lu) and the Luxembourg Parkinson’s Study.

Moreover, SMASCH contributed during the recent COVID-19 National survey for assessing Viral spread by Non-affected Carriers (CON-VINCE study)^3^ organizing appointments and results of SARS-CoV-2 PCRs and prevalence tests, as well as securing personal identifiable information (PII).

The challenge for NCER-PD program was to schedule Parkinson’s disease (PD) study participants in yearly visits, optimizing the number of appointments with different specialists from the clinical research team to happen in a certain time interval but without being too intense for the elder participants. For example, clinical data and sample(s) collection, analysis and other examinations should happen closely to be considered as a single time-point in the study. Reducing the number of visits also impacts participant retention, especially in elderly participants. Due to the special needs of some participants, both PD studies provide a mobile team to conduct examinations at different recruitment hubs across the country, thanks to a specifically equipped van. MCI-BIOME study is similar to the previous 2, taking its participants from NCER-PD and PDP cohorts.

RBD study started with a large scale survey to determine the incidence of RBD in the Luxembourgish population and increase the chances of having a sizable group to assess the link between RBD and PD. More recently, CON-VINCE study involved a wider deployment of sampling collection facilities, requiring the development of data connectors to bulk import PII from recruited participants (see Figure 2 in reference^3^). CON-VINCE study required a much intensive data and sample collection, with a bi-weekly frequency for the first 2 months (totalling 5 visits).

A more recent project includes the EU-funded ORCHESTRA project (https://orchestra-cohort.eu), which aims to establish a EU-wide cohort based on already available and new cohorts from both the EU and non-European countries (eg, Congo, Argentina) including SARS-CoV-2 infected and noninfected individuals of all ages and conditions for a retrospective evaluation and prospective follow-up. SMASCH roles include resource assignment (both infrastructure and staff), scheduling, communication, and the management and storage of PII from Luxembourg participants.

### Data protection and privacy

Above studies share a common interest to safeguard sensitive information and PII, requiring strong data protection to ensure data privacy but at the same time the possibility to exchange pseudonymized data with scientific collaborators within the framework of the General Data Protection Regulation (GDPR) of the European Union (EU). Thus, PII and clinical data are managed by 2 separate systems. Namely, all pseudonymized clinical data are stored in a dedicated REDCap^4^ instance, while SMASCH is in charge of securing PII, providing functionalities to track data modifications and access permissions (see “Features” section).

Together with the GDPR, national and institutional (eg, hospital, clinics) regulations constrain the space of solutions suitable for the aforementioned purposes. In particular, should any commercial solution be employed during such studies, it would have to comply with GDPR requirements. Importantly, as a result of the GDPR and the recent SCHREMS ruling,^5^ many institutions are reconsidering their use of cloud solutions and devising new policies, which may result in ruling out cloud services for sensitive health data. This topic is further discussed in the Supplementary Material. Besides, institutional regulations (especially public institutions) often prohibit the transfer of personal data outside their infrastructure without special agreements.

Section 2 of the Supplementary Material provides further information on SMASCH alternatives. However, all of them are closed source cloud-based solutions, with the grand majority of them only offering paid plans.

## MATERIALS AND METHODS

SMASCH follows a model view controller (MVC) architecture, a popular software design pattern that divides the program logic into 3 interconnected elements. MVC is often enforced by several web frameworks. In SMASCH, the *model* is an abstraction of the database model and manages the application state and data-related logic between the program and the relational database management system (RDBMS). The *view* comprises the presentation layer that shows the application state to the user, managing the user interface logic of SMASCH. Finally, the *controllers* interface the model and view components to handle the user actions, retrieving data from the model and rendering the response through the views.

SMASCH is written in Python 3, using Django web framework for the back-end (ie, model and controllers). For the front-end functionalities comprising the views, SMASCH employs Jinja2, a web template engine providing evaluation of Python-like expressions to enrich the rendered views. Additionally, several JavaScript libraries (eg, jQuery) drive the user interface. SMASCH can be accessed with browsers supporting ECMAScript 2015 (see Table 1) and is licensed under the open-source license AGPLv3. SMASCH code is openly available in the GitLab server from the Luxembourg Centre for Systems Biomedicine at https://doi.org/10.17881/8wvb-mt36.

**Table 1.** ECMAScript 2015—ES6 browser support

Browser	Chrome 58	Edge 14	Firefox 54	Safari 10	Opera 55
Support since	January 2017	August 2016	March 2017	July 2016	August 2018

Paginated views and application programming interfaces (APIs) allow managing very large cohorts with scant machine resources. The minimum requirements (see ”Deployment” section) allow SMASCH to handle cohort sizes up to 100K participants without issues. Bigger cohorts can be easily handled with increased resources.

## RESULTS

Close collaboration with clinical teams helped developing features aiming to reduce times of recurrent and time-demanding tasks such as generation of mail letters or finding key information. Below we describe the available features and describe the deployment procedure.

### Features

#### Permissions and provenance

SMASCH provides flexible and fine grained user permission management of the system features. Together with 2-factor authentication, it ensures restricted access to sensitive information and destructive operations. Closely related to the previous feature, provenance allows audit trail of critical modifications (eg, marking a participant as deceased) or PII export.

#### Information export

Whenever the authorized clinical team exports the list of subjects or the appointments to an Excel file, SMASCH records the user and date of the action in the system log. Of course, such an option can be disabled or limited to certain members of the clinical team with authorization to use this feature. This feature enables a user-friendly approach to export information for further use such as importing it to other medical systems or statistical tools for data analysis. The clinical team can select which fields to export, deciding whether to export PII or not. Very often, strict regulations call for different approaches involving manually supervised import/export of information between systems to prevent unnecessary access to sensitive patient/participant data in electronic health record (EHR) databases.

#### Notifications

A notification menu (see Figure 2B) warns the users about incomplete tasks (eg, missing appointments, unfinished visits) and reminds them about approaching appointments and visits in advance.

#### Redcap consistency checks

To ensure record consistency between separated systems, SMASCH implements a series of checks on demographic data (eg, birth date, vital status) using a restricted REDCap token with limited access, thus unable to access medical or clinical information. Should a mismatch occur between systems, a warning would be issued in the notification menu.

#### Sample collection summary

Different appointment types (eg, sample collection) can be registered with their corresponding items (eg, saliva kits). With this information SMASCH sends regular e-mails to biobanks and relevant providers to help forecasting stock requirements for sample collection kits or organizing pickup of collected samples.

#### User location awareness

For a tailored user experience and faster usage, members of the clinical team can select which locations (eg, clinics, hospitals, biobanks) they work on, filtering the daily planning (see Figure 2D) to the appointments happening on their locations.

#### Mail templates

To facilitate output of recurring documents, SMASCH supports uploading document templates in different languages. SMASCH can register the participant spoken languages, filtering the templates accordingly whenever a document is issued and automatically generates the appointment letters and visit vouchers in the language spoken by the study participant.

#### Custom fields

Adaptability is an essential requirement for rapid evolving scenarios. SMASCH supports custom fields of different types (eg, text, dates) to further describe a subject, visit, or appointment.

#### Extract, transform and load

Extract, transform and load pipelines (ETLs) enable the implementation of connectors both to and from other systems. Currently, such ETL procedures can pull information from different survey systems such as RedCap or Alchemer (https://www.alchemer.com/), helping to import and update subject and appointment information. Currently, SMASCH ETLs are developed as custom modules in Python.

Such ETLs enable as well interfacing systems storing EHRs. However, in practice, very often strict privacy regulations call for an intermediary to import/export records in CSV/TSV format in a supervised manner. Such measures are taken to avoid unnecessary access to sensitive data by other tools and limit the space for vulnerabilities and potential data breaches. Nevertheless, such political decisions are beyond our control, but condition how SMASCH should interface with other systems, which often implies not benefiting from the potential automatic and unsupervised capacities of certain systems in favor of other nonfunctional requirements such as security and privacy. Notwithstanding, SMASCH is looking forward to improving this feature (see “Future work” section).

The Supplementary Material and SMASCH documentation provide further information regarding the setting of such ETLs.

### Deployment

**Figure 1.**
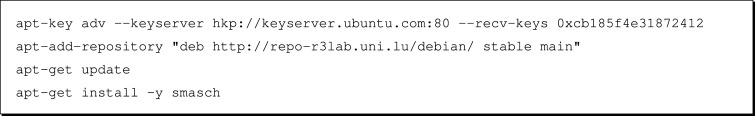
Installation of SMASCH in Debian based Linux systems.

Our production deployments of SMASCH commonly employ Linux machines, using a dedicated encrypted partition for PostgreSQL to store the system’s database. However, these deployment choices are not mandatory and SMASCH is able to work with other databases as well. The clinical studies where SMASCH is involved are long-term projects requiring equally long-term software support. For this reason the development team of SMASCH maintains its unit test coverage around 90%. Installation can be done via Debian package as described in Figure 1. Alternative installation methods (eg, Docker) are also available. For SMASCH, a virtual machine with a single CPU core and 2GB of RAM are the minimum requirements. To secure access to SMASCH, HTTPS should be enforced by the web server (eg, NginX, Apache).

In contrast to cloud-based only solutions, the provision of SMASCH source code and its corresponding Docker container facilitates the deployment of SMASCH in any infrastructure or institutional data center (eg, hospitals or clinics) that may require the use of SMASCH. And at the same time, it allows for remote deployments on cloud, as such requirement is independent of SMASCH implementation.

## DISCUSSION

Stable and well-maintained software is key for the careful management of long-term projects. In this sense, SMASCH provides a solution for MASPHs in the context of longitudinal clinical studies. The MASPH is defined as the problem of scheduling patients who need appointments on a subset of hospital or clinical resources by gathering all stakeholders in the scheduling process.^1^ In this sense, the scheduling process comprises both *combination appointments* and *appointment series*. The former entails multiple appointments for a single patient planned on the same day so that a patient requires fewer visits. The latter consists of multiple (recurring) appointments that may span several weeks or months.^6^

In turn, the long-term needs of clinical studies justify the employment of well-maintained methods, frameworks, and libraries such as Python and Django which have a proven track of continuous updates to rely on. At the same time, the high-level abstraction provided by Python and the adaptability of Djangos MVC framework enable a fast and iterative software development life-cycle that is able to cope with the rapidly evolving needs of the clinical team, reducing response times to new scenarios. The views provided by SMASCH quickly summarize the relevant information, and at the same time, are properly split to enable fine-grained permission management.

**Figure 2.**
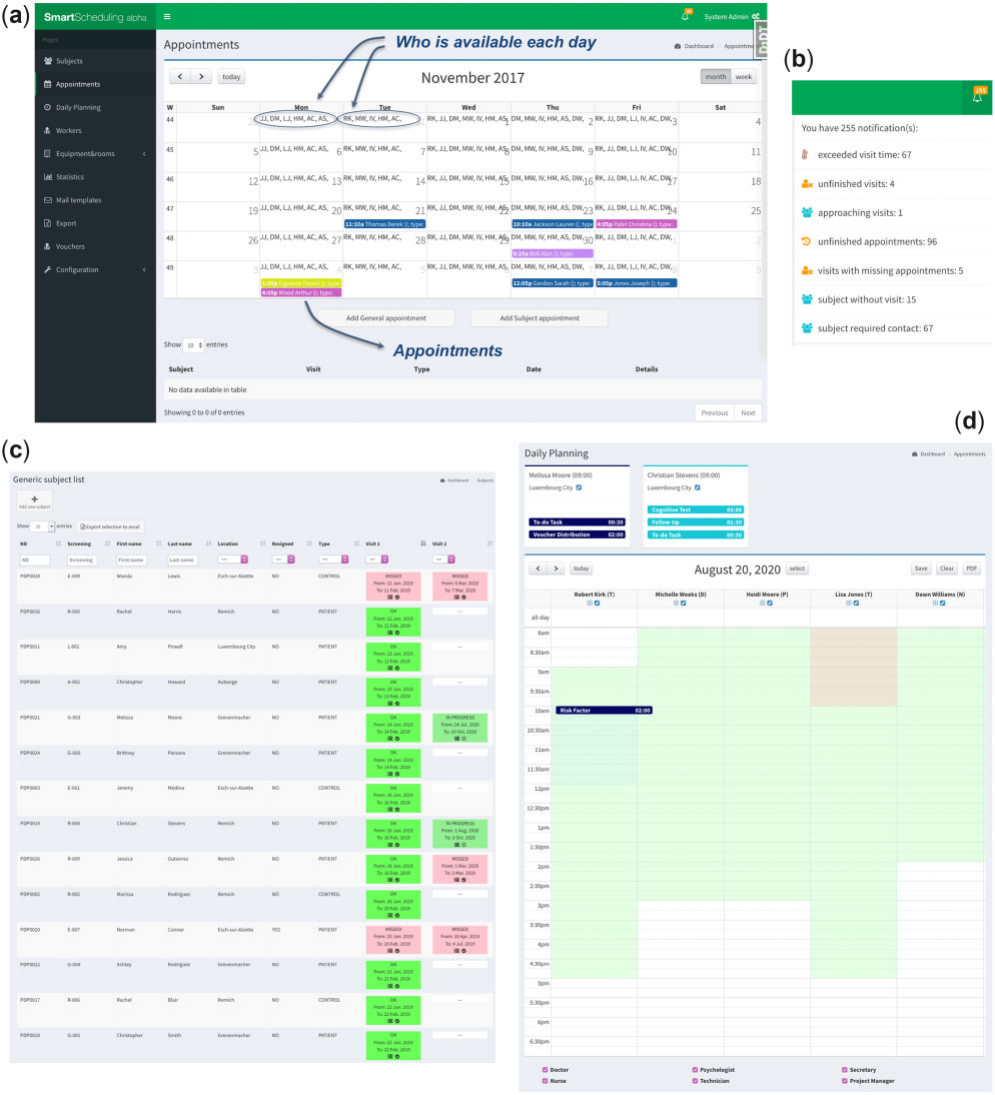
SMASCH interface and functionalities. Image (A) displays the appointment calendar with the initials indicating workers availability. Appointment colors indicate different appointment types. (B) Notification menu showing different warnings and reminders. (C) List of the study subjects (ie, participants) showing information and visit status. (D) Daily planning with the participant assessments on top which can be dragged and dropped to the calendar. Workers are sorted from left to right based on their availability. On mouse-over, the workers and participant names show a tool-tip box indicating the spoken languages to help choosing a suitable worker. Sidebar menu seen in A has been cropped out from C and D for the sake of clarity and space.

Similarly, SMASCH modular design allows interoperability with other systems through connectors, enabling its participation in multi-institutional projects that require importing and exporting information from and to different systems (see “Extract, transform and load” section). For instance, CON-VINCE study required the development of data connectors to bulk import PII from recruited participants. Figure 2 from reference^3^ depicts the role of SMASCH in the data flow from CON-VINCE. In this case, the PII from the recruited participants was sent from the survey company (TNS-Ilres) to SMASCH, where it is securely stored and accessed only by the corresponding clinical team.

### Future work

SMASCH future is open to improve the usability and interoperability with other services. Upcoming features include SMS reminders for participant appointments and the support for Lightweight Directory Access Protocol (LDAP) to facilitate multi-project access to the workers. Particularly, LDAP authentication is already available in the next release candidate. Additionally, further support for ETL procedures is currently under development aiming to increase interoperability with other data sources through a dedicated ETL API.

## CONCLUSION

In this application note, we presented Smart Scheduling, a web-based multi-appointment scheduling system designed to handle multi-appointment scheduling problems and management tasks characteristic from longitudinal clinical studies.

In general, scheduling systems can help optimizing a number of metrics that ultimately affect the success of clinical studies. Scheduling systems impact the *number of participants per consultation session* which affects participant waiting times and staff overtime. Similarly, the *length of the appointment interval* and the *number of participants per appointment slot* affect resource idle time and waiting times. Other metrics such as *participant overbooking* must be weighed too. For instance, “no-shows” may cause gaps in the schedule, increasing resource idleness. Conversely, overbooking can improve participant access times but at the cost of increasing waiting times and staff overtime.

Although SMASCH did not have the opportunity to be specifically evaluated in these regards, SMASCH has proven useful in multiple clinical research studies in which it has been actively involved since 2017. SMASCH features, such as its daily planning view, help the clinical team ponder the aforementioned factors and spot overloaded or idle agendas for a better plan of the visits, appointments, and assessments. The flexible design of SMASCH allows for a fast and iterative life cycle with high adaptability to new scenarios requiring rapid deployments, such as the recent CON-VINCE program. Consequently, SMASCH is continuously updated to fit the needs of the respective clinical research facility teams, who improved their response time and throughput thanks to a well-defined set of features coupled with a user-friendly interface. We hope SMASCH features help to sustain high participant retention and assist clinical teams in the successful development of longitudinal clinical studies.

Finally, recent regulations such as the EU’s GDPR call for strict data accountability, which together with national and institutional regulations requires further measures to ensure data privacy, especially in health-related settings. These regulations rule out the possibility of cloud-based solutions and hinder outsourcing such responsibilities. To date, we believe SMASCH is the only free and open-source scheduling system for clinical studies able to satisfy the above needs.

## FUNDING

This work was supported by the Luxembourg National Research Fund (FNR) within the National Centre of Excellence in Research on Parkinsons disease with grant number: (NCER-PD [FNR/NCER13/BM/11264123]).

## AUTHOR CONTRIBUTIONS

VS, RS, and RK conceived the idea, and collected the functional and technical requirements. PG, JL, CV, VG, and PM developed SMASCH and ES conducted software testing. VS supervised the project. CV, PG, CP, VS, RS, and RK wrote and edited the manuscript. All authors reviewed the manuscript.

## SUPPLEMENTARY MATERIAL

Supplementary material is available at *JAMIA Open* online.

## Supplementary Material

ooac038_Supplementary_Data
