# Supplementary material for: Smart Scheduling (SMASCH): multi-appointment scheduling system for longitudinal clinical research studies
Source: JAMIA Open. 2022 May 25;5(2):ooac038. doi: 10.1093/jamiaopen/ooac038 (PMC9150077; doi:10.1093/jamiaopen/ooac038)
Supplement: ooac038_Supplementary_Data [file ooac038_Supplementary_Data.zip › ooac038_Supplementary_Data/SMASCH_supp.pdf]

SMASCH: Supplementary Material

The following supplementary material describes in detail the configuration steps needed to setup a Extract, Transform & Load script in SMASCH (see § 1). Additionally, this document provides more information regarding the present alternatives to SMASCH (see § 2).

1 Extract, Transform & Load pipelines and connectors

As described in the main manuscript. Extract, transform and load pipelines (ETLs) enable the implementation of connectors both to and from other systems. Currently, such ETL procedures can pull information from different survey systems such as RedCap or Alchemer (https://www.alchemer.com/), helping to import and update subject and appointment information. Currently, SMASCH ETLs are developed as custom modules in Python. The following appendix describes how to make use of such ETLs from SMASCH interface.

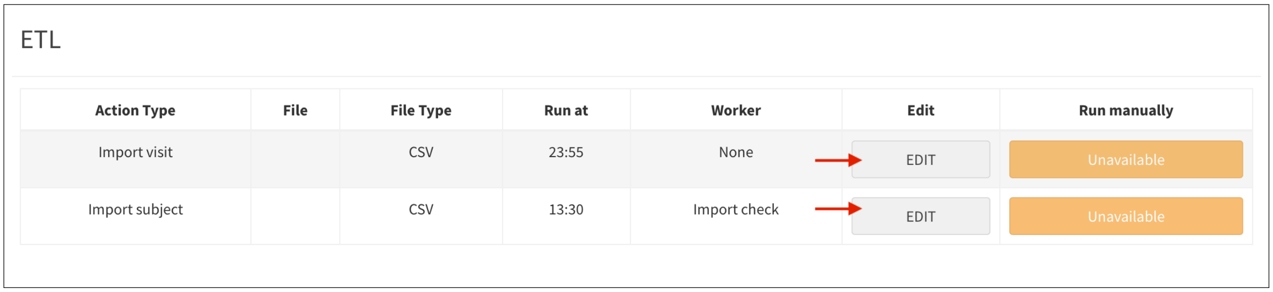

ETL

Action Type	File	File Type	Run at	Worker	Edit	Run manually
Import visit		CSV	23:55	None	EDIT	Unavailable
Import subject		CSV	13:30	Import check	EDIT	Unavailable

Fig. 1: Screenshot depicting part of the Study configuration page with the table of available ETLs. In this case, two example ETLs are shown.

From the study configuration it is possible to access the ETL settings. In the example from Figure 1, there are two ETLs that allow for the automatic or manual importing of files containing the details of subjects or appointments. Information that will be available in the Subjects / Appointments page. If the data from such files contains information about existing subject/appointment, the corresponding records in SMASCH will be updated.

The details of such ETLs can be modified by clicking the Edit button. The form fields must be filled with the same names employed in the header of the CSV import files. For instance, as seen in Figure 2, the field "First Name" will be retrieved from column `first_name`.

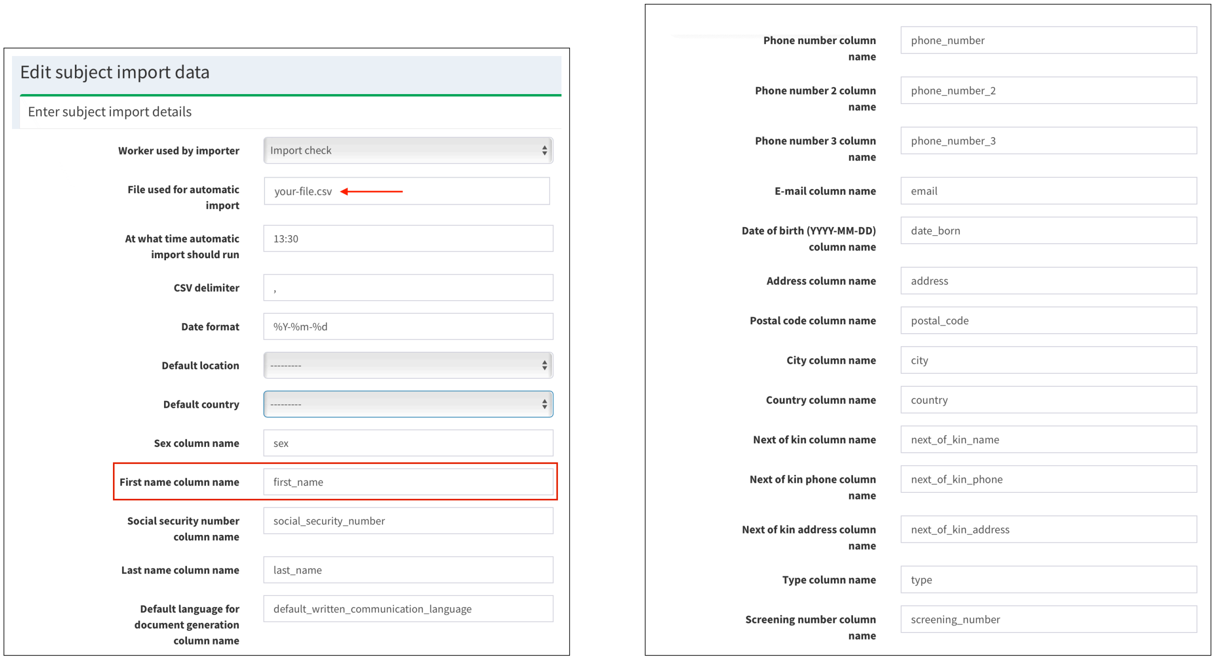

(a) Configuration form.

(b) Continuation.

Fig. 2: ETL fields configuration example.

The files can be copied to a configured directory in SMASCH machine. The user is free to choose the method of copy (e.g. FTP, SCP, rsync).

By default, the folder `/usr/lib/smasch/data/etl` will be used as ETL directory. From this folder, CSV files (see example from Figure 3) will be imported to SMASCH whenever the ETLs are manually run or automatically executed. The automatic update will be performed daily using the hour(s) provided in the field "At what time automatic import should run" (see Fig. 2).

In order to identify the subject, the import file must contain the reference to subject number or screening number. Without at least one of these columns in the CSV file, import will fail.

Further documentation regarding ETLs and other SMASCH features can be found in the following web-page: https://smasch.pages.uni.lu/.

```
first_name,last_name,city,nd_number
John,Doe,Belval,ND0099
Ian,Smith,Luxembourg,ND0100
```

Fig. 3: Example of CSV file containing records to be imported.

2 Alternative solutions

To date, the authors of this paper are not aware of similar solutions (both commercial or free) that comply with both the General Data Protection Regulation (GDPR) from the European Union (EU) and the requirements of real-world health settings (see the Data Protection and Privacy section of the manuscript). Most alternatives imply cloud-based solutions that preclude their deployment under hospital and clinical infrastructures. Importantly, as a result of the GDPR and the recent SCHREMS ruling [1], many institutions are reconsidering their use of cloud solutions and devising new policies, which may result in ruling out cloud services for sensitive health data. This topic is further discussed below. Furthermore, institutional regulations (specially public institutions) often prohibit the transfer of personal data outside their infrastructure without special agreements.

Moreover, the high price of commercial solutions together with the lack of dedicated IT personnel, leaves many low-budget clinical teams with the sole option of using spreadsheets to organise their work and collect clinical trial data [2, 3]. Open source and free solutions ease the creation of studies in projects and institutions with low resource budgets.

Table 1 summarises some of the solutions available for clinical and health management. It has been difficult for the authors to find an exhaustive list of tools similar to SMASCH. Therefore, the listed tools are rather diverse and multi-purpose, with features aimed at different domains such as physicians, clinicians, etc.

All solutions appearing in the table are cloud-based, which from the point of view of the client, eases the deployment of the solution. However, cloud-based solutions often outsource their server hosting requirements to services such as Amazon Web Services and Microsoft Azure. Such companies provide cloud hosting services with data centres located in different world regions. However, it is not clear to what extent the companies offering cloud-based clinical management services request specific locations for their hosted servers to lay within the EU borders. Half of the solutions mention GDPR on their websites in different forms.

Entities based on the European Economic Area that aim to transfer personal data to the United States of America (USA) and other non-EEA jurisdictions must provide "essentially equivalent" protections to those available in the EEA [4]. In July 2020, The Court of Justice of the European Union (CJEU) held the Schrems II decision [1]. First, the court found that USA surveillance programs do not meet the requirements of Article 52 of the EU Charter on Fundamental Rights.

Second, the court found that with respect to the USA EU data subjects lack actionable judicial redress, as required by Article 47 of the EU Charter.

Since then, the European Data Protection Supervisor (EDPS) has opened several investigations regarding the use of cloud services provided by Amazon Web Services and Microsoft [5].

In July 2018, a Portuguese hospital was fined 400.000 for infringement of the GDPR. The Portuguese Supervisory Authority (CNPD) reportedly concluded that the hospital “did not put in place appropriate technical and organisational measures to protect patient data” [6]. In February 2022, the National Commission for Computing and Liberties (CNIL[1]) of France ordered website managers to comply with the GDPR “by ceasing to use the Google Analytics functionality (under the current conditions) or by using a tool that does not involve a transfer outside the EU” [7].

Furthermore, all but one solutions are commercial, and none of them is open-source. This hinders scientific goals such as reproducibility and transparency. Proper audit and replication of studies are essential in science. In this sense, open-source code can be inspected, adapted and enhanced by researchers all over the world [8].

We believe that even though the list of tools is not exhaustive, it provides a good overview of the health software landscape and the design trends they follow. SMASCH differentiates from the above’s alternatives, providing an open-source solution that responds to the needs of real-world clinical settings.

As explained in the deployment section of the main manuscript, the provision of SMASCH source code and its corresponding Docker container facilitates the deployment of SMASCH in any infrastructure or institutional data centre (e.g. hospitals or clinics) that may require the use of SMASCH. And at the same time, it allows for remote deployments on the cloud, as such a requirement is independent of SMASCH implementation. This contrasts with the solutions presented in Table 1 that are cloud-based only.

[1]CNIL is an independent French administrative regulatory body whose mission is to ensure that data privacy law is applied to the collection, storage, and use of personal data.

TABLE 1: Summary table of available solutions providing health management services. The symbols ✓and ✗ stand for yes and no, respectively.

	Commercial	Cloud-based	GDPR support	Free option	Open-Source
Care Patron [9]	✓	✓	Their website mentions support for GDPR.	Free plan available.	✗
DocMeIn [10]	✗	✓	No mention about GDPR.	Completely Free.	✗
PetalHealth [11]	✓	✓	Their website mentions support for GDPR.	✗	✗
LumaHealth [12]	✓	✓	No mention about GDPR.	✗	✗
Veribook [13]	✓	✓	No mention about GDPR.	✗	✗
PracticeSuite [14]	✓	✓	No mention about GDPR.	✗	✗
ClinicSoftware [15]	✓	✓	Their website mentions support for GDPR.	✗	✗
PatientManager [16]	✓	✓	Their website mentions support for GDPR.	✗	✗
hCue [17]	✓	✓	No mention about GDPR.	✗	✗
Jane [18]	✓	✓	Their website mentions support for GDPR.	✗	✗

References

[1] C-311/18, data protection commr v. facebook ire. ltd. & schrems [2020] [schrems ii], paras. 105, 162, at 31, 40. URL https://curia.europa.eu/juris/document/document.jsf?docid=228677&text=&doclang=EN&pageIndex=0&cid=1175857.

[2] Oracle health Services. Moving beyond Excel to purpose-built applications for speedier study startup. https://web.archive.org/web/20220328153354/https://www.oracle.com/a/ocom/docs/dc/em/dmo400002773_oracle_whitepaper_moving%20beyond%20excel%20to%20purpose-built%20applications%20for%20speedier%20ssu_15aug2019.pdf. Accessed: 2022-03-28.

[3] Devin Incerti, Howard Thom, Gianluca Baio, and Jeroen P Jansen. R you still using Excel? the advantages of modern software tools for health technology assessment. *Value in Health*, 22(5):575–579, 2019.

[4] Joseph Liss, David Peloquin, Mark Barnes, and Barbara E Bierer. Demystifying schrems ii for the cross-border transfer of clinical research data. *Journal of Law and the Biosciences*, 8(2):lsab032, 2021.

[5] The edps opens two investigations following the schrems ii judgement. URL https://edps.europa.eu/press-publications/press-news/press-releases/2021/edps-opens-two-investigations-following-schrems_en.

[6] Portuguese hospital receives and contests 400,000 fine for gdpr infringement. URL https://web.archive.org/web/20210422010509/https://www.natlawreview.com/article/

portuguese-hospital-receives-and-contests-400000-fine-gdpr-infringement.

[7] Use of google analytics and data transfers to the united states: The cnil orders a website manager/operator to comply. URL https://www.cnil.fr/en/use-google-analytics-and-data-transfers-united-states-cnil-orders-website-manageroperator-comply.

[8] Darrel C Ince, Leslie Hatton, and John Graham-Cumming. The case for open computer programs. *Nature*, 482(7386):485–488, 2012.

[9] Care patron. URL https://www.carepatron.com/.

[10] Docme in. URL https://www.docmein.com/.

[11] Petal health. URL https://www.petal-health.com/.

[12] Luma health. URL https://www.lumahealth.io/.

[13] Veribook. URL https://veribook.com/.

[14] Practice suite. URL https://practicesuite.com/.

[15] Clinic software. URL https://clinicsoftware.com/.

[16] Patient manager. URL https://patientmanager.eu/.

[17] hCue. URL https://www.myhcue.com/.

[18] Jane. URL https://jane.app/.
